# *Oleum ocimi gratissimi* as a promising natural preservative against fish spoilage bacteria through i006Ehibition of planktonic growth and biofilm formation

**DOI:** 10.1016/j.fochx.2025.102816

**Published:** 2025-07-19

**Authors:** Yan Li, Yi Yu, Yiwei Cui, Luyi Jiang, Yongkang Luo, Hongkai Xie, Hui Hong

**Affiliations:** a College of Biology and Environmental Engineering, Zhejiang Shuren University, Hangzhou 310015, China; b College of Food Science and Engineering, Nanjing University of Finance and Economics, Nanjing 210023, China; c College of Food Science and Nutritional Engineering, China Agricultural University, Beijing 100083, China

**Keywords:** Antimicrobial, Biofilm formation, Fish spoilage bacteria, *Oleum ocimi gratissimi*

## Abstract

Strategies to control spoilage bacteria and biofilm formation are essential for minimizing food losses. Although *Oleum ocimi gratissimi* (OG) effectively preserves chill-stored fish, its specific inhibitory mechanisms against fish-derived spoilage bacteria remain unclear. This study investigated the antibacterial activity of OG against *Aeromonas sobria*, *Pseudomonas versuta*, and *Shewanella putrefaciens*. OG exhibited minimum inhibitory concentrations (MIC) of 2, 0.75, and 0.375 mg/mL and minimum bactericidal concentrations (MBC) of 3, 4, and 3 mg/mL against these bacteria, respectively. At MIC levels, OG inhibited planktonic growth by disrupting cell membranes, inducing adenosine triphosphate (ATP) leakage, reducing ATPase activity, and altering cellular morphology. Biofilm formation was completely suppressed, with OG further diminishing metabolic activity and extracellular polysaccharide/protein. These findings elucidate the dual role of OG in inhibiting bacterial growth and biofilm formation, highlighting its potential to enhance fish safety and shelf life by targeting biochemical and physiological pathways in spoilage bacteria.

## Highlights

• OG had strong antimicrobial and anti-biofilm activity against spoilage bacteria.
• OG against planktonic growth of spoilage bacteria by multiple mechanisms.
• OG reduces the proportion of polysaccharides and proteins in the biofilm.
• OG is promising as a good natural preservative.

## 1 Introduction

Food loss is receiving growing global attention due to its impact on both food security and sustainability. Globally, nearly 40 % of meat products were lost throughout the supply chain, such as production, processing, transportation, and storage (United Nations Environment Program, 2024), causing food waste and economic loss. Fish flesh is considered among the most perishable foods, particularly chill-stored fish, as their rich protein and high moisture content are suitable for microbial growth. During the supply chain, specific spoilage bacteria are considered the main contributors to the deterioration of meat product quality (Morales et al., 2016). In food systems, planktonic specific spoilage bacteria attach to food surfaces by producing extracellular polymeric substances (EPS) such as proteins and polysaccharides, gradually forming biofilms (Rumbaugh & Sauer, 2020). These biofilms serve as protective barriers, shielding the bacteria from environmental stresses. This protection enhances bacterial survival and spreads, further accelerating food quality deterioration (Gong et al., 2024). Therefore, there is a pressing need to develop effective intervention methods for specific spoilage bacteria.

Regardless of geographical origin, the microbial community during the spoilage of chill-stored freshwater fish is primarily composed of *Pseudomonas* spp. and *Shewanella putrefaciens* (Gram & Huss, 1996). Another report found *Pseudomonas* spp. and *Aeromonas* spp. were the specific spoilage bacteria of contain-cultured fish, while *S. putrefaciens* was not observed (Li, Jia, et al., 2020). The specific spoilage bacteria could be a little varies according to different packaging methods, storage temperatures, and processing techniques (Doulgeraki et al., 2012). But at the point of chill-stored freshwater fish spoilage, *Pseudomonas* spp. is the predominant bacterium, with *Shewanella* spp. and *Aeromonas* spp. also commonly serving as dominant spoilage genera (Tan et al., 2023; Zhuang et al., 2021). The presence of specific spoilage bacteria leads to quality issues in chill-stored fish, including texture softening, discoloration, and the development of off-flavor (Li, Zhuang, et al., 2020; Zhuang et al., 2023). Additionally, the growth of *Pseudomonas* spp., *Shewanella* spp., and *Aeromonas* spp. accelerates the breakdown of umami components, such as inosinate, which negatively affects the original flavor of fish (Chen et al., 2024; Li et al., 2021). Specifically, *S. putrefaciens*, *Pseudomonas versuta,* and *Aeromonas sobria* contribute to inosinate degradation, while *S. putrefaciens* and *P. versuta* also promote the accumulation of bitter compounds like hypoxanthine in fish flesh (Li, Zhuang, et al., 2020). Therefore, management of *Pseudomonas* spp., *Shewanella* spp., and *Aeromonas* spp. during the processing and storage period is the primary approach to minimizing chill-stored fish losses.

At present, a wide variety of essential oils are used as bio-preservatives to retard the specific spoilage bacteria contamination in chill-stored fish, such as cinnamon essential oil and rosemary essential oil (Hao et al., 2021). Clove basil (*Ocimum gratissimum L*.) is a plant of the genus *Basil* in the family Labiatae. Some studies have shown that *Oleum ocimi gratissimi* (OG) possesses excellent antibacterial activities against *Staphylococcus aureus*, *Escherichia coli*, *Salmonella Typhimurium*, *Shigella flexneri*, *Pseudomonas aeruginosa*, and *Bacillus cereus* (Chimnoi et al., 2018; Melo et al., 2019; Talabi & Makanjuola, 2017). Our previous study proved that 0.5 % and 1 % concentrations of OG inhibited the growth of *Pseudomonas* spp., *Aeromonas* spp., and *Shewanella* spp. in the chill-stored bream fillets, and prolonged the shelf-life for 2–4 days (Li et al., 2023). However, few studies have been carried out on the antibacterial and antibiofilm effects of OG against fish spoilage bacteria *Pseudomonas* spp., *Aeromonas* spp., and *Shewanella* spp. The mechanisms of OG against these bacteria are poorly understood, which limits the utilization of OG for the preservation of refrigerated fish.

Therefore, this study provides new insights into the dual inhibitory effects of OG on the planktonic growth and biofilm formation of *P. versuta*, *A. sobria*, and *S. putrefaciens*. The minimum inhibitory concentrations (MIC) and minimum bactericidal concentrations (MBC) values of OG for these spoilage bacteria were quantified, and their correlation with membrane damage, energy metabolism disruption, and biofilm inhibition was examined. An effort was made to develop a new effective spoilage bacteria inhibitor, providing new solutions for food loss.

## 2 Materials and methods

### 2.1 Preparation of strains

*A. sobria*, *P. versuta*, and *S. putrefaciens* were isolated by Li, Zhuang, et al. (2020) as specific spoilage bacteria from aerobic chilled blunt snout bream fillets. In brief, A total of 84 strains were isolated from spoiled fish flesh that was stored at 4 °C for 8 days using the plate streak method, and identified through 16S rRNA gene sequencing. The dominant strains *P. versuta* (16/84), *S. putrefaciens* (12/84), and *A. sobria* (11/84) were selected and preserved in the laboratory. Strains were vacuum freeze-dried with skimmed milk powders and stored at −80 °C. The bacterial powder was shake-cultured (100 rpm) in 25 mL tryptic soy broth (Beijing Aobox Biotechnology, Co. Ltd., Beijing, China) for 12 h at 30 °C (approximately 10^7^ CFU/mL) before use.

### 2.2 Preparation of sterile blunt snout bream juice

Farmed blunt snout bream (weight: 0.96 ± 0.02 kg, length: 39.77 ± 0.51 cm) were purchased from Beijing local market and delivered alive to the laboratory. The fish were dispatched by a blow to the head. The entire procedure followed the Guidance on Treating Experimental Animals developed by China's Ministry of Science & Technology in 2006 and Regulations issued by China State Council in 1988. Then, breams were scaled, gutted, sliced into fillets, and cleaned with water. The backbone bream flesh was excised without red flesh, skinned, and minced. The flesh was mixed with an equal weight of deionized water and boiled for 10 min with sustained stirring. After cooling to room temperature, the mixture was filtered through six layers of gauze. The fish flesh juice was collected and sterilized at 121 °C for 15 min, and then stored at 4 °C before use.

### 2.3 Screening of essential oils

Thirteen spice-derived essential oils including clove (*Syzygium aromaticum*), rosemary (*Rosmarinus officinalis*), mugwort (*Artemisia argyi*), thyme (*Thymus vulgaris*), clove basil (*Ocimum gratissimum L*.), lemon peel (*Citrus limon*), lemon eucalyptus (*Corymbia citriodora*), turmeric (*Curcuma longa*), anise (*Pimpinella anisum*), grape seed (*Vitis vinifera*), carrot seed (*Daucus carota*), cumin (*Cuminum cyminum*), and black pepper (*Piper nigrum*) were sourced from Jiangxi Hengcheng Co., Ltd. (Jiangxi, China). To conduct the screening, 100 μL suspensions of *A. sobria*, *P. versuta*, and *S. putrefaciens* were spread on plate count agar (Beijing Aobox Biotechnology, Co. Ltd., Beijing, China) and allowed to dry at room temperature, respectively. A sterilized circular filter paper with a diameter of 6 mm was placed in the center of each agar plate, and 5 μL of the respective essential oil solution was applied onto the filter paper. The plates were then inverted and incubated at 30 °C for 24 h, after which, the diameter of the inhibition zone (DIZ) surrounding the filter paper was measured.

### 2.4 The effect of OG on the planktonic growth of specific spoilage bacteria

#### 2.4.1 Determination of MIC and MBC

The MIC and MBC of OG against *A. sobria*, *P. versuta*, and *S. putrefaciens* were determined through the broth dilution method according to Kang et al. (2018). OG was diluted with sterile blunt snout bream juice to achieve concentrations ranging from 0.125 to 8 mg/mL. The *A. sobria*, *P. versuta*, and *S. putrefaciens* were inoculated into each diluted concentration of juice, respectively, and shake-incubated at 30 °C for 24 h. The MIC value was the lowest concentration of OG with no visible bacterial growth in the fish juice. The MBC value was the lowest concentration of OG without bacterial growth on plate count agar (Beijing Aobox Biotechnology, Co. Ltd., Beijing, China).

#### 2.4.2 Growth curve assay

The growth curves of three bacteria were measured according to Kang et al. (2020) with some modifications. OG was diluted with sterile blunt snout bream juice to achieve the concentrations of MIC, 1/2 MIC, and 1/4 MIC. The *A. sobria*, *P. versuta*, and *S. putrefaciens* were inoculated into each diluted concentration of juice and sterile blunt snout bream juice without OG, respectively. Then, the juices were shaken at 30 °C. The growths of three bacteria were monitored by a Multiskan Spectrum (UV-2600, Unico, Shanghai, China) at 600 nm at 2 h intervals.

#### 2.4.3 Fourier transform infrared spectroscopy analysis

The *A. sobria*, *P. versuta*, and *S. putrefaciens* suspensions were treated with OG (Control and MIC) at 30 °C for 2 h. The suspensions were centrifugated and the cells were collected. Then, the cells were freeze-dried and ground with KBr (1:100; *w*/w) for Fourier transform infrared spectroscopy (FT-IR) analysis (Spectrum 1000, PerkinElmer, Massachusetts, USA). The FT-IR spectra of samples between 4000 and 400 cm^−1^ with a resolution of 4 cm^−1^ were measured.

#### 2.4.4 Determination of intracellular and extracellular ATP concentrations and ATPase activity

Intracellular and extracellular ATP concentrations were determined according to Kang et al. (2020) with some modifications. In brief, the *A. sobria*, *P. versuta*, and *S. putrefaciens* suspensions were treated with OG (Control and MIC) at 30 °C for 0.5 h. Then, the bacterial suspensions were centrifuged at 5000 rpm for 5 min. The supernatant was collected for extracellular ATP analysis, while the pellet was used for intracellular ATP and ATPase extraction. Ultrasound treatment was used to pellet on ice to release intracellular ATP. After centrifugation at 5000 rpm for 5 min, the supernatant was collected and kept on ice to minimize ATP degradation. Both intra- and extracellular ATP levels were measured using an ATP assay kit (Beyotime Bioengineering Institute, Shanghai, China) according to the manufacturer's instructions. The Na^+^ K^+^ and Ca^2+^ Mg^2+^ -ATPase were extracted using ultrasound treatment on ice without centrifugation. The Na^+^ K^+^ and Ca^2+^ Mg^2+^ -ATPase activities were then assayed through the ATPase assay kit (Jiancheng Bioengineering Institute, Nanjing, China) according to the manufacturer's instructions.

#### 2.4.5 Scanning electron microscopy analysis

The suspensions of *A. sobria*, *P. versuta*, and *S. putrefaciens* were treated with OG (Control and MIC) at 30 °C for 4 h. Bacterial cells were collected and washed with PBS three times. Bacterial suspensions were diluted and dropped onto silicon wafers. To immobilize bacteria, the silicon wafers were dried at 37 °C and soaked in 2.5 % glutaraldehyde for 2 h at 4 °C, then the cells were dehydrated gradually in graded ethanol (30, 50, 70, 90, and 100 %) for 10 min per concentration. After air-drying, the cells were gold-coated and observed under a scanning electron microscope (SEM, S—3000H, Hitachi, Tokyo, Japan).

### 2.5 The effect of OG on the biofilm

#### 2.5.1 Growth of specific spoilage bacteria biofilm

Before the quantitative detection of biofilm, safranin staining was used for qualitative analysis of the presence of biofilms. Then, the biofilm growths of *A. sobria*, *P. versuta*, and *S. putrefaciens* were quantitatively measured in a 96-well polystyrene plate (JET Biofil, Canada) through the crystal-violet staining method (Kang et al., 2020). The 20 μL of *A. sobria*, *P. versuta*, and *S. putrefaciens* suspensions were supplemented with 200 μL of bream fish juice and added to a 96-well polystyrene plate, respectively. The specific spoilage bacteria biofilms were cultured at 30 °C for 30 h, and quantitative detection of biofilms was performed every 6 h.

#### 2.5.2 Biofilm bacterial metabolic activity analysis

The biofilms of *A. sobria*, *P. versuta*, and *S. putrefaciens* were established in a 96-well polystyrene plate and cultured with OG (Control and MIC) at 30 °C for 24 h. Then, the culture medium was removed, and biofilms were washed with PBS three times. The metabolic activity of bacteria in the biofilms was evaluated through the MTT assay kit (Sangon, Shanghai, China).

#### 2.5.3 Main EPS components analysis

The biofilms of *A. sobria*, *P. versuta*, and *S. putrefaciens* were established with OG in a six-well polystyrene plate (JET Biofil, Canada) as described in 2.5.1. The main EPS components, including extracellular polysaccharides, protein, and DNA were extracted according to Kang et al. (2018) and Vazquez-Armenta et al. (2018). Briefly, after washing biofilms with PBS, 1 mL of physiological saline solution was added to each well. The biofilms were ultrasonically dispersal and disrupted for 15 min and then centrifuged at 5000 rpm for 20 min. The supernatants were collected. Detection of extracellular polysaccharides was carried out through the anthrone-H_2_SO_4_ reagent. The extracellular proteins were measured by using a micro-BCA protein assay kit (Sangon, Shanghai, China). The extracellular DNA was extracted through a Bacterial DNA Isolation Kit (Bomaide, Beijing, China) with some modifications and measured by a UV–visible spectrophotometer (UV-2600, Unico, Shanghai, China) at 260 nm absorbance.

#### 2.5.4 Morphological characteristics analysis of bacterial biofilm

*A. sobria*, *P. versuta*, and *S. putrefaciens* were incubated with OG in a six-well polystyrene plate. One piece of sterile, polished zinc sheet (10 mm × 10 mm × 0.2 mm) was added to each well and used for biofilm adhesion. Biofilms were incubated at 30 °C for 24 h and washed with PBS three times. The fixation and dehydration of biofilms on the zinc sheets were processed as described in 2.4.5. The biofilms were gold-coated and observed under a SEM (S—3000H, Hitachi, Tokyo, Japan).

### 2.6 Statistical analysis

All experiments were done in triplicate, except for the morphological characteristics analysis. Differences among all groups were analyzed through SPSS 20.0 software (SPSS Inc., Armonk, NY, USA). An analysis of the Duncan test at a confidence level of 0.05 was performed on the data.

## 3 Results and discussion

### 3.1 Antibacterial activity of OG against bacteria

#### 3.1.1 Essential oils selection

Thirteen spice-derived essential oils were selected for their diverse bioactive compounds and antimicrobial properties, allowing for an effective screening against fish spoilage bacteria. The DIZ for the thirteen EOs is presented in Table 1. The results indicated that clove essential oil had the strongest inhibitory effect against *A. sobria*, with a DIZ of 40.12 mm, followed by OG at 33.84 mm. For *P. versuta* and *S. putrefaciens*, OG exhibited the highest inhibitory activity, with DIZ values of 11.80 and 35.57 mm, respectively. According to Li, Zhuang, et al. (2020), *P. versuta* and *S. putrefaciens* exhibit stronger growth, odor production, and protein degradation abilities in fish flesh compared to *A. sobria*. Therefore, the OG, which showed the most effective inhibition against *P. versuta* and *S. putrefaciens*, was selected for further antibacterial evaluation.

**Table 1:** The inhibition zone of 13 essential oils against *P. versuta*, *S. putrefaciens*, and *A. sobria*.

Essential oils	Species	Inhibition zone (mm)		
		*A. sobria*	*P. versuta*	*S. putrefaciens*
Clove	*Syzygium aromaticum*	40.12 ± 0.39^Fc^	9.55 ± 0.47^Da^	32.35 ± 1.76^Eb^
Rosemary	*Rosmarinus officinalis*	16.60 ± 0.43^Cc^	8.09 ± 0.52^BCa^	12.60 ± 0.35^Bb^
Mugwort leaf	*Artemisia vulgaris L.*	10.26 ± 0.77^Ab^	6.13 ± 0.09^Aa^	14.07 ± 0.98^Bc^
Thyme	*Thymus mongolicus Ronn*	22.00 ± 0.46^Db^	8.47 ± 0.92^Ca^	26.89 ± 4.78^Dc^
Clove basil	*Ocimum gratissimum L.*	33.84 ± 0.97^Eb^	11.80 ± 0.36^Ea^	35.57 ± 3.06^Eb^
Lemon peel	*Citrus Limonum*	13.7 ± 0.75^Bb^	7.40 ± 0.06^Ba^	12.46 ± 0.69^Bb^
Lemon Eucalyptus	*Eucalyptus citriodora Hook.f.*	21.04 ± 0.76^Db^	7.91 ± 0.08^Ba^	23.96 ± 0.85^Db^
Ginger	*Zingiber officinale Roscoe*	NA	NA	7.29 ± 0.26^Aa^
Star anise	*Illicium verum*	10.86 ± 0.46^Aa^	NA	NA
Grape seed	*Vitis vinifera L.*	NA	NA	NA
Carrot seed	*Daucus carota*	NA	NA	NA
Cumin	*Cuminum cyminum L.*	NA	NA	19.70 ± 0.35^Ca^
Black pepper	*Piper nigrum*	NA	NA	NA

Note: The inhibition zone included the diameter of disk (6 mm). Results are presented as mean ± standard deviation. The same capital letters in a column indicate no significant differences (*p* > 0.05). The same lowercase letters in a row indicate no significant differences (*p* > 0.05).

The chemical compositions of OG as reported by Li et al. (2023) are presented in Supplementary Table S1. Eugenol (76.78 %) is the most predominant component, followed by β-caryophyllene (14.83 %) and α-humulene (3.21 %). Eugenol, β-caryophyllene, and α-humulene are natural food additives that have excellent antioxidant and antimicrobial activity (Chappell & Robert, 2020; Ullah et al., 2023; Zheng et al., 2019). Eugenol exerts an antibacterial effect mainly by disrupting the cytoplasmic membrane, leading to increased permeability, ion leakage, and cell lysis. It also induces oxidative stress, alters membrane fatty acid composition, and inhibits key bacterial enzymes, collectively impairing cellular function and viability (Marchese et al., 2017). Di Pasqua et al. (2007) reported that eugenol treatment significantly altered the membrane fatty acid composition in *Pseudomonas fluorescens*, while its effects on *A. sobria*, *P. versuta*, and *S. putrefaciens* remain insufficiently studied.

#### 3.1.2 MIC and MBC

Table 2 illustrates the inhibitory effects of various concentrations of OG on *A. sobria*, *P. versuta*, and *S. putrefaciens*. The MICs for *A. sobria*, *P. versuta*, and *S. putrefaciens* were 0.375, 2, and 0.75 mg/mL, respectively. The MBCs were 3, 4, and 3 mg/mL, respectively. These findings also proved that the sensitivity of these bacteria to OG varies, with *P. versuta* being the least sensitive. The antimicrobial efficacies of EOs are predicated on their ability to alter or penetrate the cell membranes of microorganisms. Some components of EOs act on the microbial cell membrane, causing it to rupture and leading to microbial inactivation. Other components may penetrate the cell membrane, interfere with the internal functions of bacteria, and ultimately cause their demise. Di Pasqua et al. (2006) observed that adding eugenol to the *Pseudomonas* culture medium did not significantly change the fatty acid profile of the cell membrane. The lipopolysaccharides in the outer membrane of *Pseudomonas* might partially block the eugenol, preventing it from reaching the cell membrane. The findings of this study align with previous research and may help explain the relatively lower inhibitory effect of OG on *P. versuta*. In this study, the inhibitory effects of OG on three specific spoilage bacteria exhibit a concentration-dependent relationship. Overall, it demonstrated strong antimicrobial activity against *A. sobria*, *P. versuta*, and *S. putrefaciens*.

**Table 2:** The MIC and MBC values of *Oleum ocimi gratissimi* against *P. versuta*, *S. putrefaciens*, and *A. sobria*.

*Oleum ocimi gratissimi* (mg/mL)	Bacteria species		
	*A. sobria*	*P. versuta*	*S. putrefaciens*
16	−	−	−
12	−	−	−
8	−	−	−
6	−	−	−
4	−	−	−
3	−	+	−
2	+	+	+
1.5	+	++	+
1	+	++	+
0.75	+	++	+
0.5	+	++	++
0.375	+	++	++
0.25	++	++	++
0.1875	++	++	++
0.125	++	++	++
0.09375	++	++	++
0.0625	++	++	++
0.046875	++	++	++
0.03125	++	++	++
0.0234375	++	++	++
0.01565	++	++	++
0.007825	++	++	++

Note: “++” indicates visible growth of bacteria in tubes; “+” indicates no visible growth of bacteria in tubes; “-” indicates no growth of bacteria on agar medium for plate counting.

#### 3.1.3 Growth curve assay

**Fig. 1:**
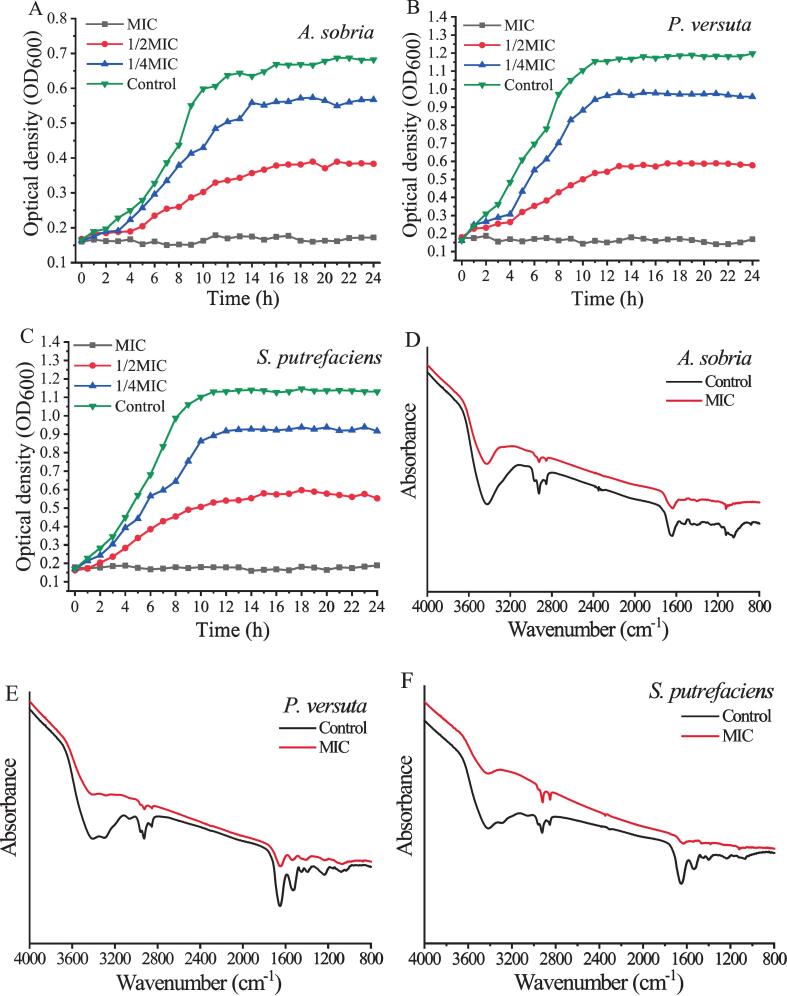
The growth curve of *A. sobria* (A), *P. versuta* (B), and *S. putrefaciens* (C) treated with Oleum ocimi gratissimi. FT-IR spectra of *A. sobria* (D), *P. versuta* (E), and *S. putrefaciens* (F) cells treated with Oleum ocimi gratissimi.

The effect of OG on the growth curves of *A. sobria*, *P. versuta*, and *S. putrefaciens* is shown in Fig. 1. After 14 h, the growth of *A. sobria* in the control group reached a stable phase (Fig. 1A). In the presence of 1/4 MIC and 1/2 MIC of OG, the growth of *A. sobria* decreased by 22 % and 44 %, respectively. Similar inhibitory effects were observed for *P. versuta* and *S. putrefaciens*. For *P. versuta*, growth decreased by approximately 17 % and 50 % in the 1/4 MIC and 1/2 MIC groups, respectively (Fig. 1B). In the case of *S. putrefaciens*, growth decreased by 20 % and 53 % in the 1/4 MIC and 1/2 MIC groups, respectively (Fig. 1C). Furthermore, complete growth inhibition of all three bacteria was achieved by OG at MIC within the 24-h culture period.

### 3.2 OG-induced destruction in bacterial cells

#### 3.2.1 Cellular components

The effects of OG on the cellular components of three bacteria were investigated using FT-IR spectra. Numerous studies have confirmed the effectiveness of FT-IR in monitoring alterations in microbial cell membrane composition (Moghimi et al., 2017; Zoumpopoulou et al., 2010).

Fig. 1D shows the FT-IR analysis of *A. sobria* cells treated with OG at the MIC level. Significant reductions were observed in the absorption peaks at 2959 cm^−1^, 2925 cm^−1^, and 2854 cm^−1^. Typically, the spectral region between 3000 and 2800 cm^−1^ is characterized by vibrational features of cell membrane fatty acids, specifically at 2960 cm^−1^, 2925 cm^−1^, and 2860 cm^−1^ (Alvarez-Ordóñez et al., 2011). This reduction indicated damage to membrane lipid components in *A. sobria*. Decreased absorbance was also noted at 1650 cm^−1^, 1550 cm^−1^, 1455 cm^−1^, and 1400 cm^−1^. These peaks correspond to proteins, peptides, ester groups, and nucleic acids within the 1800–1300 cm^−1^ region, including the amide I band at 1650 cm^−1^ and amide II band at 1550 cm^−1^ (Al-Qadiri, Al-Holy, et al., 2006). The decrease of these peaks suggested degradation of structural proteins. Additionally, the absorption peak at 1080 cm^−1^ was nearly absent. The spectral region between 1300 and 900 cm^−1^ includes signals from cell wall components, cell membranes, and nucleic acids (Sundaram et al., 2012). This region features a nucleic acid peak at 1080 cm^−1^ and carbohydrate peaks at 1000–950 cm^−1^ (Goodacre et al., 1996; Lu et al., 2011). The decrease of this peak indicated damage to nucleic acids in *A. sobria*.

For *P. versuta* (Fig. 1E), the control group exhibited distinct absorption peaks at 2961 cm^−1^, 2923 cm^−1^, and 2863 cm^−1^, which were reduced in the OG-treated group. The peak at 2960 cm^−1^ nearly disappeared, indicating a disruption of fatty acids in *P. versuta* cell membranes. Moreover, there were reductions in the absorption intensities of the amide I band at 1650 cm^−1^, amide II band at 1550 cm^−1^, and structural protein peaks at 1451 cm^−1^ and 1393 cm^−1^, suggesting damage to cell structural proteins. Furthermore, the characteristic phosphodiester peak at 1231 cm^−1^ was nearly absent, indicating severe disruption of the phospholipid bilayer structure. The nucleic acid peak at 1082 cm^−1^ showed a slight decrease, suggesting interference with nucleic acid synthesis. These findings are consistent with a previous study, which reported that eugenol can bind to bacterial DNA, impairing replication and transcription processes (Cui et al., 2008).

In Fig. 1F, the FT-IR analysis of *S. putrefaciens* treated with MIC OG showed a decrease in the characteristic absorption peak at 2960 cm^−1^, with slight reductions at 2919 cm^−1^ and 2851 cm^−1^. Similar reductions were observed in the absorption intensities of the amide I band at 1650 cm^−1^, the amide II band at 1550 cm^−1^, and structural protein peaks at 1451 cm^−1^ and 1393 cm^−1^, indicating damage primarily to proteins and the phospholipid bilayer structure.

These findings suggest that OG disrupts the cell membrane structure, proteins, and nucleic acids of *A. sobria*, *P. versuta*, and *S. putrefaciens*, thereby impacting the normal physiological activities of three specific spoilage bacteria.

#### 3.2.2 Intra- and extracellular ATP and cell membrane ATPase

**Fig. 2:**
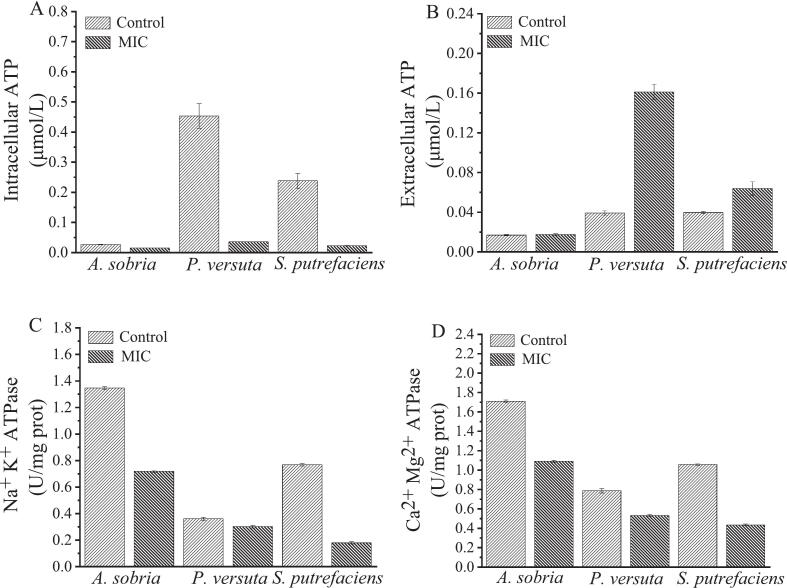
The effect of *Oleum ocimi gratissimi* on the intracellular ATP concentration (A), extracellular ATP concentration (B), Na^+^ K^+^ ATPase (C), and Ca^2+^ Mg^2+^ ATPase (D) of *A. sobria*, *P. versuta*, and *S. putrefaciens*.

ATP is the direct source of energy for physiological activities such as biosynthesis and material transportation of specific spoilage bacteria. ATP and ATPase play an important role in maintaining the normal physiological functions of specific spoilage bacteria. As shown in Fig. 2A and Fig. 2B, OG influenced the intra- and extracellular ATP concentrations of three bacteria. The intracellular ATP concentrations in the OG-treated groups of *P. versuta* and *S. putrefaciens* were decreased compared to the control group, whereas the extracellular ATP concentrations of OG-treated *P. versuta* and *S. putrefaciens* exceeded the control group, increasing 4.1 and 1.6 times, respectively. The results indicated that the MIC OG altered the cell membrane permeability of *P. versuta* and *S. putrefaciens*, leading to the leakage of intracellular ATP. While in the *A. sobria* group, the intracellular ATP concentration was slightly lower than the control group. In the meantime, the extracellular ATP concentration remains almost unchanged. Fig. 2C and Fig. 2D present two typical ATPase activities that are essential for cellular life activities. After OG-treatment, the activities of Na^+^ K^+^ ATPase in *A. sobria* and *S. putrefaciens* were decreased. At the same time, the activities of Ca^2+^ Mg^2+^ ATPase were reduced in the *A. sobria*, *P. versuta*, and *S. putrefaciens* groups. Na^+^ K^+^ ATPase and Ca^2+^ Mg^2+^ ATPase are crucial transmembrane enzymes on the cell membranes of specific spoilage bacteria. Na^+^ K^+^ ATPase is responsible for exchanging sodium and potassium ions, maintaining a low sodium and high potassium state within the cell (Kumari & Rathore, 2020). Ca^2+^ Mg^2+^ ATPase helps regulate intracellular levels of calcium and magnesium, which are essential for various physiological functions, such as cell movement (King et al., 2020). The results suggest that OG induced the death of three bacteria by disrupting energy metabolism and inhibiting the activities of Na^+^ K^+^ ATPase and Ca^2+^ Mg^2+^ ATPase.

#### 3.2.3 Morphological changes

**Fig. 3:**
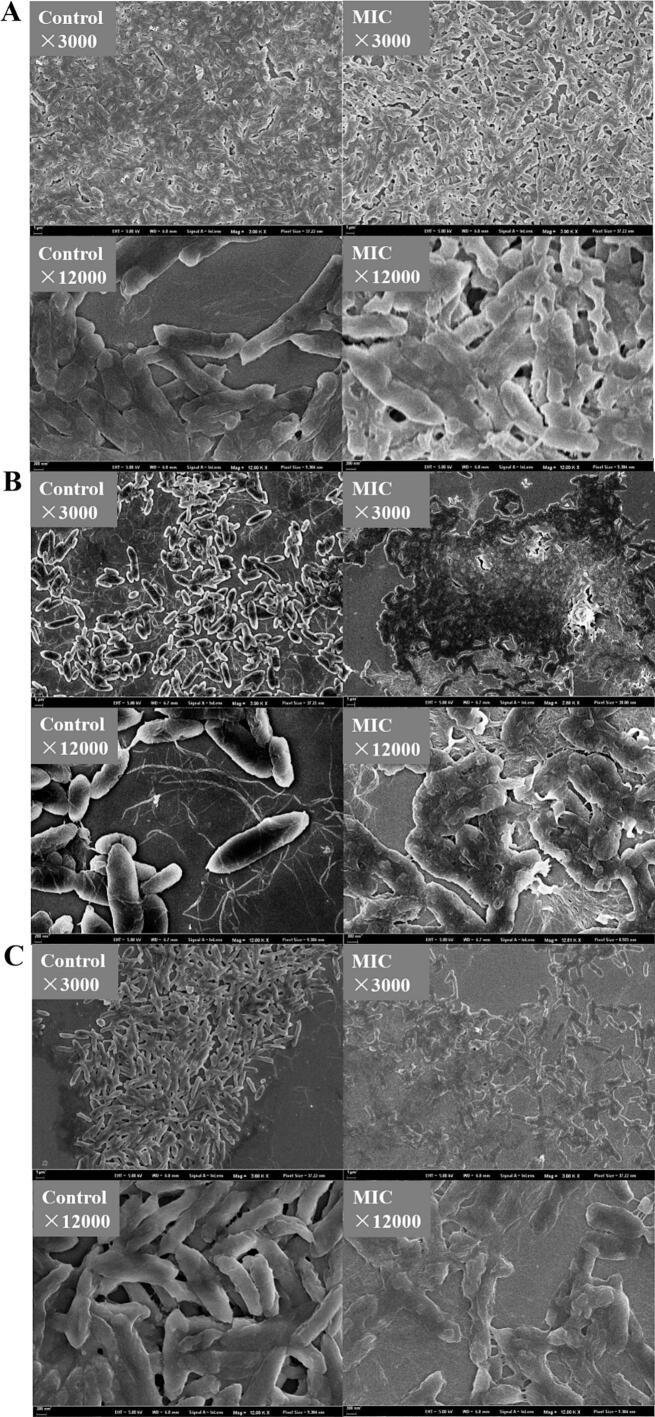
The SEM images of *A. sobria* (A), *P. versuta* (B), and *S. putrefaciens* (C) treated with MIC level of *Oleum ocimi gratissimi*.

SEM was used to observe the surface morphological changes between the control and OG-treated *A. sobria*, *P. versuta*, and *S. putrefaciens* cells. As shown in Fig. 3A, untreated *A. sobria* exhibited a typical Gram-negative structure with a complete and well-defined elongated shape. Following treatment with OG at the MIC, the cells showed membrane damage, appearing flattened with visible pores and partial lysis. In the *P. versuta* group (Fig. 3B), membrane disruption was even more pronounced in the OG-treated *P. versuta* cells. While the control *P. versuta* cells appeared full and rounded with a short rod shape, OG-treated cells were severely damaged, showing significant membrane disintegration. Fig. 3C illustrates the morphological changes in *S. putrefaciens*. Compared to the control group, the MIC-treated bacterial cells exhibited structural collapse and membrane destruction. In contrast to *A. sobria*, no distinct membrane pores were observed in *P. versuta* and *S. putrefaciens*. Instead, their cell membranes showed extensive disruption and detachment, indicating severe structural damage. These morphological differences suggest that OG may exert species-specific effects on membrane integrity, potentially due to differences in cell envelope composition or stress response mechanisms. Overall, SEM analysis confirmed that OG disrupted bacterial membrane integrity, which contributes to the inhibition of bacterial planktonic growth and biofilm formation.

### 3.3 Effect of OG on bacteria biofilm formation

#### 3.3.1 Biofilm formation of three bacteria

**Fig. 4:**
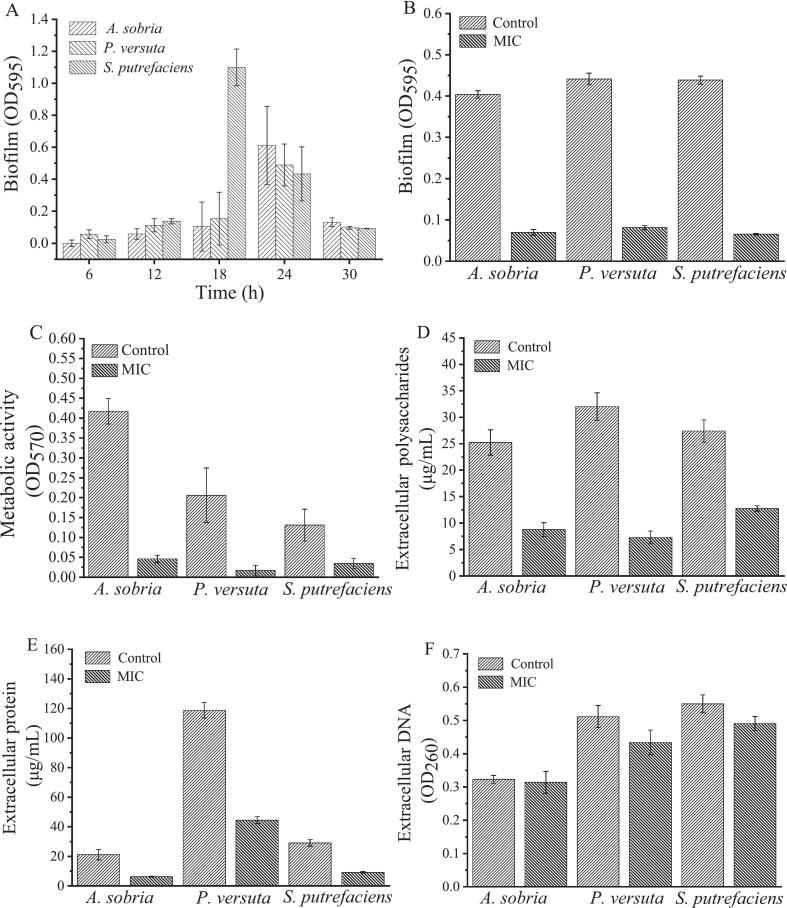

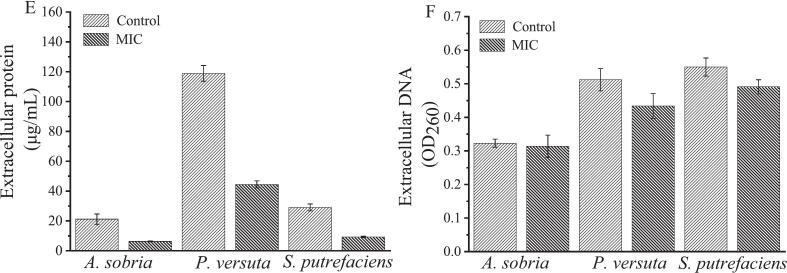
The biofilm formation (A) of *A. sobria*, *P. versuta*, and *S. putrefaciens*; The biofilm formation (B), metabolic activity (C), extracellular polysaccharides (D), extracellular proteins (E), and extracellular DNA (F) of *A. sobria*, *P. versuta*, and *S. putrefaciens* treated with MIC level of *Oleum ocimi gratissimi*.

The safranin staining experiment confirmed that all three types of spoilage bacteria produce biofilms (Supplementary Fig. 1). As shown in Fig. 4A, the total amount of biofilms increased over time, with *S. putrefaciens* peaking at 18 h. For *A. sobria* and *P. versuta*, biofilm formation continued to increase until 24 h. *S. putrefaciens* exhibited an earlier peak in biofilm formation compared to the other two bacteria. This can be attributed to its ability to produce high levels of cyclic diguanylate, a signaling molecule that promotes biofilm formation. Additionally, its nitrate-reducing respiratory activity contributes to the acceleration of biofilm development on certain surfaces. Then, all three bacteria showed a significant reduction in biofilm formation at 30 h. Based on these observations, a 24-h incubation period was chosen for subsequent experiments.

#### 3.3.2 Biofilm formation and bacterial metabolism

Fig. 4B illustrates the biofilm formation of the three bacterial strains following treatment with OG at the MIC level. In the control group, the optical density at OD595 values of biofilm formation for *A. sobria*, *P. versuta*, and *S. putrefaciens* were 0.40, 0.44, and 0.44, respectively. After treatment with MIC OG, the OD595 values decreased to 0.07, 0.08, and 0.07, respectively. Bacterial biofilm formation is closely related to food spoilage and is also a mechanism of auto-aggregation and persistent food contamination (Gong et al., 2024; Yi et al., 2022). In this study, OG effectively inhibited the biofilm formation of *A. sobria*, *P. versuta*, and *S. putrefaciens*, which may help reduce their colonization and spoilage potential in fish products.

The metabolic activities of three bacteria in biofilms were assessed using the MTT colorimetric method. This method is based on the presence of mitochondrial succinate dehydrogenase in living microbial cells, which reduces MTT to form insoluble formazan crystals that accumulate in the cells. These formazan deposits can then be dissolved with dimethyl sulfoxide for measurement. As shown in Fig. 4C, the metabolic activities of *A. sobria*, *P. versuta*, and *S. putrefaciens* were 0.42, 0.21, and 0.13, respectively. Compared to the control group, the metabolic activity of three bacteria decreased after treatment with MIC OG. The metabolic activities of *A. sobria*, *P. versuta*, and *S. putrefaciens* decreased by 89.0 %, 91.8 %, and 73.6 %, respectively. OG significantly reduced the bacterial metabolic activity in the biofilms of three bacteria, especially *A. sobria* and *P. versuta*.

#### 3.3.3 Biofilm composition

The changes in the extracellular polysaccharides, proteins, and DNA of biofilms formed by three bacteria are shown in Fig. 4. After 24 h of cultivation, *A. sobria*, *P. versuta*, and *S. putrefaciens* formed extracellular polysaccharides at levels of 25.2, 32.0, and 27.4 μg/mL, respectively (Fig. 4D). Treatment with MIC OG reduced these levels to 8.8, 7.3, and 12.8 μg/mL, representing decreases of 2.86, 4.38, and 2.14 times, respectively. Extracellular polysaccharides play a crucial role in biofilm formation and protect bacteria from inhibitory substances. OG inhibits the formation of extracellular polysaccharides from three bacteria, thereby disrupting biofilm formation and exerting antibacterial effects. Fig. 4E displays changes in extracellular proteins, which constitute the main matrix of bacterial biofilms. In the control group, *A. sobria*, *P. versuta*, and *S. putrefaciens* produced extracellular proteins at levels of 21.2, 118.8, and 29.0 μg/mL, respectively. In the OG-treated group, these levels decreased to 6.2, 44.5, and 9.2 μg/mL, respectively. OG inhibited the production of extracellular proteins. The inhibition of extracellular proteins weakens the structural integrity and cohesion of the biofilm matrix, disrupting its three-dimensional architecture. Additionally, since many extracellular proteins are involved in adhesion, enzymatic activity, and signaling, their reduction may impair the functional stability of the biofilm, making it more susceptible to external stress and antimicrobial agents. Fig. 4F demonstrates changes in extracellular DNA. Different from extracellular polysaccharides and proteins, the reductions in extracellular DNA contents in the OG-treated groups were not significant. However, the contents of extracellular DNA in the *P. versuta* and *S. putrefaciens* groups appear to show reductions. The inhibition of extracellular DNA disrupts biofilm structural integrity by weakening the physical network that stabilizes the matrix and facilitates cell-to-cell adhesion. These findings indicated that OG effectively inhibits the secretion of extracellular polysaccharides and proteins by *A. sobria*, *P. versuta*, and *S. putrefaciens*, with the most pronounced effect observed on extracellular proteins in *P. versuta*.

#### 3.3.4 Morphological changes

**Fig. 5:**
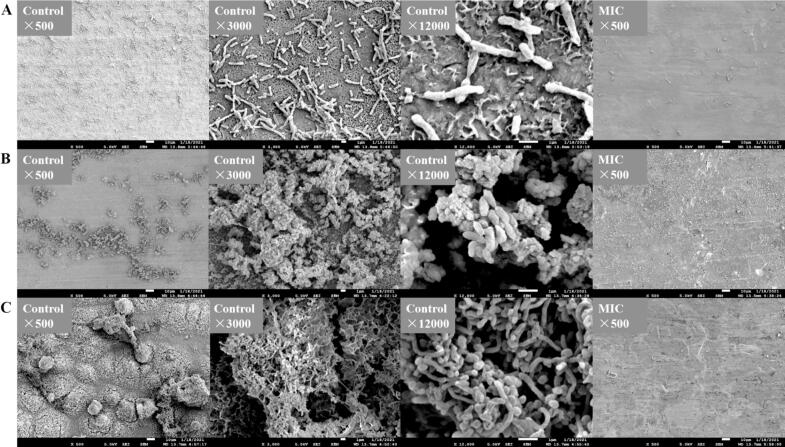
The SEM images of *A. sobria* (A), *P. versuta* (B), and *S. putrefaciens* (C) biofilm and biofilm treated with MIC level of *Oleum ocimi gratissimi*.

Microscopic and SEM observations revealed that *A. sobria*, *P. versuta*, and *S. putrefaciens* form biofilms both on glass and zinc, each displaying distinct morphologies. These specific biofilm structures are illustrated in Supplementary Fig. 2 and Fig. 5, respectively. The biofilm morphology of *A. sobria* was characterized by a flat appearance with loosely wrapped external layers, closely packed cells, and minimal stacking. Supplementary Fig. 2B and Fig. 5B illustrate the biofilm morphology of *P. versuta*, which features stacked and elevated structures with short rod-shaped cells. Extracellular compounds are prominently visible, intertwined with bacteria, and stacked to form the biofilm. The biofilm morphology of *S. putrefaciens* resembles *P. versuta* with stacked and elevated structures. The cells of *S. putrefaciens* were elongated, surrounded by an external wrapping, and exhibited a distinct mesh-like internal structure. The cells are densely packed within this mesh-like arrangement, contributing to the biofilm formation. After treatment with MIC OG, no biofilm structures were observed for *A. sobria*, *P. versuta*, and *S. putrefaciens*. Additionally, viable cells of *A. sobria*, *P. versuta*, and *S. putrefaciens* were nearly absent in the results of both the Microscopic and SEM. These results indicate that MIC OG effectively inhibits biofilm formation by *A. sobria*, *P. versuta*, and *S. putrefaciens*.

## 4 Conclusion

The present study demonstrated that OG effectively inhibits planktonic growth and biofilm formation in *A. sobria*, *P. versuta*, and *S. putrefaciens*. At the MIC level, OG exposure nearly abolished bacterial growth, disrupted cell morphology, reduced metabolic activity, and inhibited the secretion of extracellular polysaccharides and proteins, resulting in minimal biofilm development. These findings confirm OG as a promising natural preservative for controlling fish-derived spoilage bacteria. Unlike synthetic preservatives, which may raise consumer health concerns, OG offers a naturally derived alternative with potent antimicrobial effects comparable to conventional treatments. While this study focused on individual spoilage bacteria, future research should investigate the efficacy and mechanisms of OG against complex spoilage microbiota to support its broader application in food preservation.

## Author contribution

Yan Li: Methodology, Carried out the experiment, Data curation, Writing-original draft, Project administration, Funding acquisition. Yi Yu: Carried out the experiment, Data curation. Yiwei Cui: Carried out the experiment. Luyi Jiang: Carried out the experiment. Yongkang Luo: Writing-review & editing. Hongkai Xie: Conceptualization, Writing-review & editing. Hui Hong: Conceptualization, Writing-review & editing.

## CRediT authorship contribution statement

**Yan Li:** Writing – original draft, Methodology, Funding acquisition, Data curation. **Yi Yu:** Data curation. **Yiwei Cui:** Data curation. **Luyi Jiang:** Data curation. **Yongkang Luo:** Writing – review & editing. **Hongkai Xie:** Writing – review & editing, Conceptualization. **Hui Hong:** Writing – review & editing, Conceptualization.

## Declaration of competing interest

The authors declare that they have no known competing financial interests or personal relationships that could have appeared to influence the work reported in this paper.

## Data Availability

The authors do not have permission to share data.

## Appendix A Supplementary data

Supplementary material Image 1
