# Supplementary material for: Oleum ocimi gratissimi as a promising natural preservative against fish spoilage bacteria through i006Ehibition of planktonic growth and biofilm formation
Source: Food Chem X. 2025 Jul 19;29:102816. doi: 10.1016/j.fochx.2025.102816 (PMC12311605; doi:10.1016/j.fochx.2025.102816)
Supplement: Supplementary file 1 — Supplementary material [file mmc1.docx]

**Table S1.** Chemical compositions of *Oleum ocimi gratissimi*.

| Essential oil | Compositions | Proportion (%) |
| --- | --- | --- |
| *Oleum ocimi gratissimi* | Eugenol | 76.78 |
|  | β-Caryophyllene | 14.83 |
|  | α-Humulene | 3.21 |

Note: data adapted from Li et al. (2023).

**
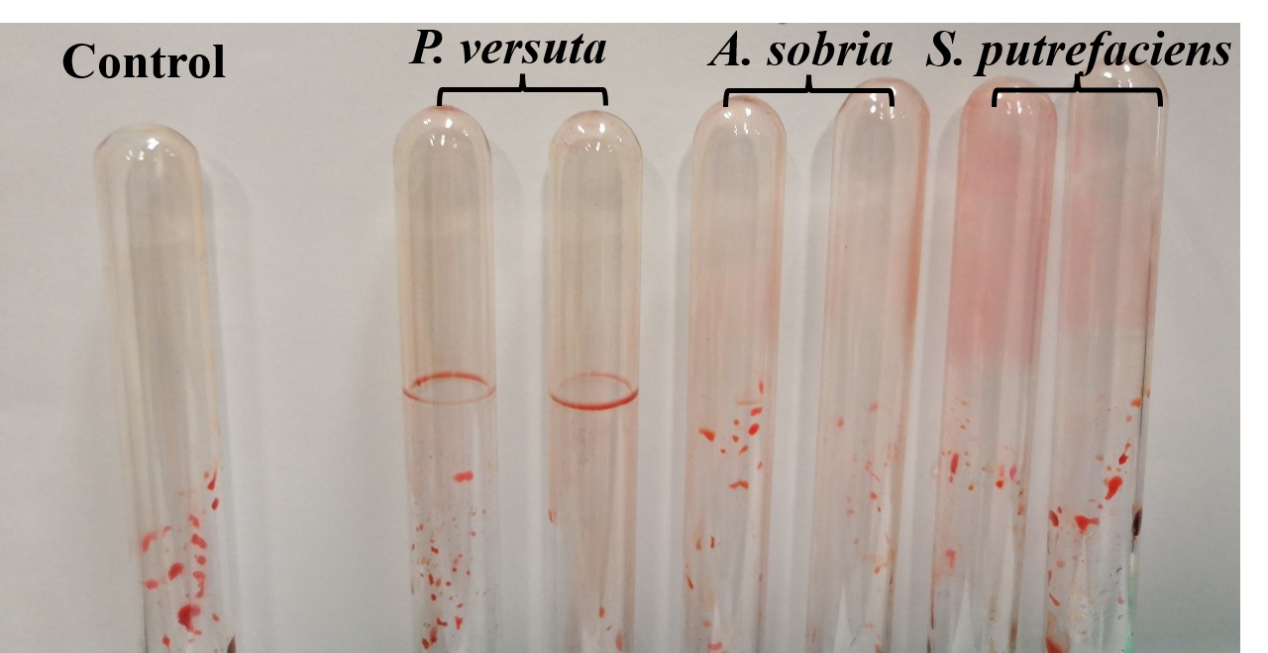
Fig.S1** Safranin-stained photo of biofilms formed by *P. versuta*, *A. sobria*, and *S. putrefaciens*.


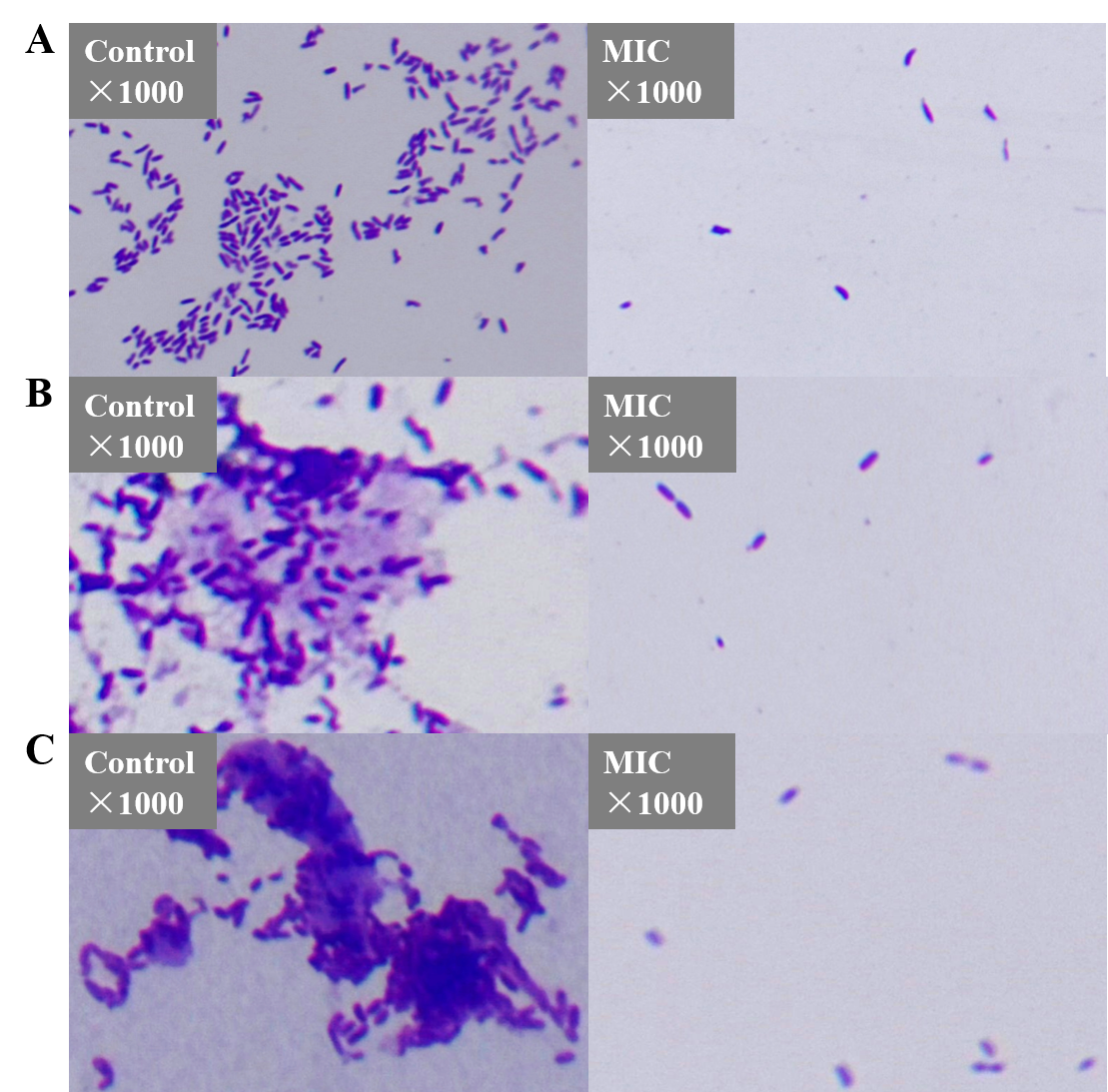


**Fig. S2** The microscope images of *A. sobria* (A), *P. versuta* (B), and *S. putrefaciens* (C) biofilm and biofilm treated with MIC concentrations of *Oleum ocimi gratissimi*.

**Reference**:

Li, Y., Zhang, L., Zhuang, S., Li, D., Hong, H., Lametsch, R., Tan, Y., & Luo, Y. (2023). Shelf life extension of chilled blunt snout bream fillets using coating based on chia seed gum and *Oleum ocimi gratissimi*. Food Bioscience, 54, Article 102853. https://doi.org/10.1016/j.fbio.2023.102853.
